# European Union Reference Laboratories support the National food, feed and veterinary Reference Laboratories with rolling out whole genome sequencing in Europe

**DOI:** 10.1099/mgen.0.001074

**Published:** 2023-07-25

**Authors:** Valeria Michelacci, Adrien Asséré, Simone Cacciò, Marina Cavaiuolo, Kirsten Mooijman, Stefano Morabito, Susanne Karlsmose Pedersen, Maroua Sayeb, Bo Segerman, Magnus Simonsson, Hanna Skarin, Rosangela Tozzoli, Angela van Hoek, Rene Sjøgren Hendriksen

**Affiliations:** ^1^​ European Union Reference Laboratory for Escherichia coli including Shiga toxin-producing E. coli, Department of Food Safety, Nutrition and Veterinary Public Health, Istituto Superiore di Sanità, Rome, Italy; ^2^​ European Union Reference Laboratory for Listeria monocytogenes, Laboratory for Food Safety, Anses, Maisons-Alfort, France; ^3^​ European Union Reference Laboratory for Parasites, Department of Infectious Diseases, Istituto Superiore di Sanità, Rome, Italy; ^4^​ European Union Reference Laboratory for Coagulase Positive Staphylococci, Laboratory for Food Safety, Anses, Maisons-Alfort, France; ^5^​ European Union Reference Laboratory for Salmonella, National Institute for Public Health and the Environment, Bilthoven, Netherlands; ^6^​ European Union Reference Laboratory for Antimicrobial Resistance, Technical University of Denmark, National Food Institute, WHO Collaborating Center for Antimicrobial Resistance in Food borne Pathogens and Genomics, FAO Reference Laboratory for Antimicrobial Resistance, Kgs. Lyngby, Denmark; ^7^​ European Union Reference Laboratory for Campylobacter, National Veterinary Institute, Uppsala, Sweden; ^8^​ European Union Reference Laboratory for Foodborne Viruses, Swedish Food Agency, Uppsala, Sweden

**Keywords:** bioinformatic analysis, capacity building, EURL, Europe, next generation sequencing, NRL

## Abstract

The Inter European Union Reference Laboratories (EURLs) Working Group on Next Generation Sequencing (NGS) involves eight EURLs for microbiological food and feed hazards and has been working since 2017 to promote the adoption of NGS by the National Reference Laboratories (NRLs) in the European Union. This work illustrates the results of the first 5 years of activity. By working together, the EURLs involved have released guidance documents for assisting NRLs in all the steps of NGS, helping the transition from classical molecular methods towards whole genome sequencing while ensuring harmonization, with the final aim of improving preparedness in the use of NGS to characterize microbial hazards and trace the sources of infection.

## Data Summary

The authors confirm all supporting data, code and protocols have been provided within the article.

**Impact Statement**

The Inter European Union Reference Laboratories (EURLs) Working Group on Next Generation Sequencing (NGS) involves eight EURLs for microbiological food and feed hazards and has been active since 2017 on a mandate of the European Commission for promoting the adoption of NGS across the respective National Reference Laboratory (NRL) networks in the European Union, by building capacity and ensuring harmonization of procedures. Here, we present the main achievements of the working group at the current stage and plans for the future, mainly consisting of offering continuously updated resources as guidance documents, reference genome sequences and training opportunities, contributing to the growing ability of the NRLs to apply NGS technology for diagnosing and/or tracing microbiological foodborne infectious diseases in Europe.

## Introduction

The global surveillance and outbreak investigation of infectious disease agents, including viral, parasitic and bacterial pathogens, and associated antimicrobial resistance (AMR) in a One Health context is currently amid a paradigm shift from traditional microbiology to genomics. The transition started around 2010 with the introduction of affordable benchtop next-generation sequencing (NGS) platforms. A decade later, this technology represents a powerful all-in-one solution to obtain complete characterization of microbial pathogens at lower costs and often in shorter time than with traditional microbiology. However, as with all molecular methods, its adoption in routine monitoring of infectious disease agents in food and animals and in outbreak investigations aimed at source tracking requires optimization and harmonization.

The European Union Reference Laboratories (EURLs) for food and feed hazards are designated according to Regulation (EU) 2017/625 of the European Parliament [1], also stating the tasks of the EURLs, which include the improvement and harmonization of the analytical methods to be applied by official laboratories, provision to the National Reference Laboratories (NRLs) in EU countries with details, as well as guidance on such methods and coordination of their application through regular organization of inter-laboratory comparative studies. Regulation (EU) 2017/625 also states that each Member State shall designate one or more NRLs for each EURL, in order to coordinate the activities of the official laboratories with a view to harmonizing and improving the methods of laboratory analysis. This organization is based on the transfer of knowledge from the European level to the Member State level and is critical to build preparedness to respond in a harmonized way to events that could emerge in the area of foodborne hazards.

In 2017, the ‘Inter-EURLs Working Group (WG) on NGS’ was established based on a mandate by the European Commission (EC), with the aim to promote the use of NGS across the EURLs’ networks, by building capacity and ensuring harmonization of procedures within the EU. The WG consists of representatives of eight EURLs for the following microbiological hazards: *Escherichia coli* including Shiga-toxin-producing *E. coli *, *Listeria monocytogenes *, coagulase-positive staphylococci, *Salmonella *, *Campylobacter *, foodborne parasites, AMR and foodborne viruses. The location of the EURLs involved in the WG is detailed in Table 1. The WG operates in liaison with the work of the European Food Safety Authority (EFSA) and the European Centre for Disease Prevention and Control (ECDC), based on the NGS mandate sent by the EC for the implementation of a ‘One Health system for the collection and analysis of whole genome sequencing (WGS) data from human and food/animal isolates to ECDC and EFSA’ (M-2020-0015, EFSA-Q-2020-00101) [2], while ensuring no overlap of activities between the entities involved.

**Table 1.** List of European Union Reference Laboratories for microbiological hazards

European Union Reference Laboratories for microbiological hazards	Location
European Union Reference Laboratory for *Escherichia coli* including Shiga toxin-producing *E. coli *	Istituto Superiore di Sanità, Department of Food Safety, Nutrition and Veterinary Public Health, Rome, Italy
European Union Reference Laboratory for *Listeria monocytogenes *	Anses, Laboratory for Food Safety, Maisons-Alfort, France
European Union Reference Laboratory for Coagulase-Positive Staphylococci	Anses, Laboratory for Food Safety, Maisons-Alfort, France
European Union Reference Laboratory for *Salmonella *	National Institute for Public Health and the Environment, Bilthoven, the Netherlands
European Union Reference Laboratory for *Campylobacter *	National Veterinary Institute, Uppsala, Sweden
European Union Reference Laboratory for Foodborne Parasites	Istituto Superiore di Sanità, Department of Infectious Diseases, Rome, Italy
European Union Reference Laboratory for Antimicrobial Resistance	Technical University of Denmark, National Food Institute, Kgs Lyngby, Denmark
European Union Reference Laboratory for Foodborne Viruses	Swedish Food Agency, Uppsala, Sweden

The WG meets twice a year, with the aim of coordinating activities and providing guidance documents for the organization of NGS-based proficiency tests (PTs), for the application of NGS laboratory procedures and analytical bioinformatics tools and for benchmarking analytical methods, while following the activities for the development of ISO 23418 : 2022 ‘Whole genome sequencing for typing and genomic characterization of bacteria’ [3]. Additionally, common strategies for training activities to steer capacity building for the NRLs in the EU are deployed and reference materials for benchmarking of tools are provided. The outcomes of the WG activities are uploaded and routinely updated on the web pages dedicated to these activities of each of the involved EURLs. Here, we describe in detail the accomplishments by the WG in the implementation of NGS among the food and animal NRLs in the EU.

## NGS adoption across National Reference Laboratories

The level of adoption of NGS across the NRLs was estimated by launching a survey in spring 2018 and subsequently by monitoring the participation in the PTs for NGS-based methods organized by the EURLs [4]. About half of the NRLs participating in the survey reported as having access to NGS technology, mainly used on a selection of isolates rather than on a routine basis. Limited funding and lack of specific expertise were perceived as the main hindrances by the NRLs still not having access to NGS. Nevertheless, great interest in participating in future PTs and training on NGS organized by the EURLs was expressed in all the networks, stressing the relevance of the activities of the EURLs and the WG. In the last 4 years, the picture has generally remained stable, with some EURLs reporting around 20 NRLs having access to and being able to use NGS. Generally, NGS is more widely adopted for bacterial species compared with viruses and parasites, where NGS is often hindered by a limited amount of nucleic acids as starting material and requires the optimization of *in vitro* amplification steps.

In detail, EURLs for coagulase-positive staphylococci, parasites and foodborne viruses reported ongoing progress throughout their networks since 2018, when fewer than ten NRLs per network had declared as having access to NGS. This improvement was mainly achieved due to participation in research projects, which are expected to provide tangible preparedness in the next couple of years.

## Harmonization of procedures

The use of established protocols in the production and the analysis of NGS data is essential to achieve comparability of results, which is particularly important for example in the framework of an outbreak investigation. With the aim of supporting the NRLs, since 2020 the Inter-EURLs WG on NGS has released and routinely updated a collection of guidance documents, as detailed below. All these documents are published on the web pages specifically dedicated to the activities of the WG, present on the websites of each of the involved EURLs. An example is represented by the page included in the EURL for *E. coli* website [5], linking all the released documents.

### NGS laboratory procedures

The availability of high-quality nucleic acids (DNA/RNA) is a prerequisite for the successful application of NGS methodologies. Due to the large biological differences among bacterial, viral and parasitic pathogens, different methods are required, in particular for uncultivable target organisms. A guidance document was prepared by gathering Standard Operating Procedures (SOPs) generated during previous EU projects, such as COMPARE [6], ENGAGE [7] and INNUENDO [8], and/or from the work of the EURLs. The document comprises protocols for DNA and RNA extraction, which are applicable to specific pathogens or specific matrices, as well as combined protocols and complete workflows [9]. Additionally, a supporting document for preparing high-quality DNA for whole genome sequencing was released, describing the main control steps to be applied when extracting the DNA from bacterial pathogens and the criteria to be used to assess DNA quality and concentration [10].

### Bioinformatics tools for basic characterization and cluster analysis

The bioinformatics tools most used for NGS data analyses, including quality checking, trimming, assembly, multi-locus sequence typing (MLST), virulotyping, serotyping and identification of AMR genes, were referenced in a guidance document [11]. In addition to open-source stand-alone tools, commercial software was also listed, as well as webservers offering easy-to-use solutions for several steps of the analysis to shield the end-user from complexities.

A further guidance document that aimed to inform and support NRLs in the choice of methods to be used for cluster analysis was released [12]. This document includes descriptions of methods and tools available for cluster analysis of WGS data, both SNP and gene-by-gene approaches, and for visualization of the results. The document also presents comparisons between the different approaches and the possible impact they can have on the interpretation of results.

### Quality control support

Standardization of WGS procedures from DNA preparation to the final genome is pivotal to provide reliable data for surveillance and outbreak detection. To ensure the production of reliable high-quality genomic data, laboratories routinely applying NGS technology should participate in inter-laboratory studies or external quality assurance schemes (EQAs)/PTs. A document describing the approach used in genomics PTs at each of the EURLs and the experiences collected during the organization was published and will be routinely updated [13]. The EURLs for *E. coli *, *L. monocytogenes *, *Salmonella *, *Campylobacter* and AMR all performed PTs focusing on prediction of: (1) specific subtypes (e.g. serotype, MLST); (2) determinants (e.g. virulence factors, acquired AMR determinants including chromosomal point mutations, plasmid replicons); (3) phylogeny based on SNPs or gene-by-gene approaches; and (4) genome quality metrics.

In the future, the organization of PTs focusing on metagenomics approaches could be useful for the networks dealing with difficult-to-grow hazards, such as viruses and parasites.

Different approaches were used to collect the PT results, in some cases requiring the submission of sequence files, in addition to analysed results, which poses problems of heavy data exchange and of workload for the analysis needed at the EURLs for evaluation. In other cases, the analysed results are submitted without exchanging sequence files, which provides an easier solution, though not allowing control of the quality of the sequence file(s) produced.

### Reference whole genome sequences

A reference genome collection, useful for the validation and benchmarking of bioinformatics tools, was compiled. Sequences of different pathogens produced by participants of PTs organized by the EURLs are listed in the guidance document [14].

### Wet and dry benchmarking

EURLs and NRLs need to document the performance of the laboratory procedures and bioinformatics tools with appropriate and comprehensive benchmarking. The document ‘Guidance document for WGS-Benchmarking’ [15] was prepared to present the approach elaborated upon to perform benchmarking of the different NGS steps. It features a checklist for bacterial genomic analysis using NGS technology that sets standards for the analytical ‘wet bench’ process and for bioinformatics or ‘dry bench’ analyses of sequence data.

The wet bench component includes any of the following processes: handling of strains, extraction of nucleic acids, fragmentation, barcoding (molecular indexing) of isolates, enrichment of targets for exome or gene panels, adapter ligation, amplification, library preparation, flow cell loading and generation of sequence reads. Sequence generation is almost entirely automated, and the output consists of millions to billions of short sequence reads. The dry bench workflow is followed by intensive computational and bioinformatics analyses that use a variety of algorithms to align the short sequence reads to a linear reference bacterial genome sequence, and to perform *de novo* assembly. The document also includes statistical analyses to perform the benchmarking.

### Training and capacity building

As NGS practices are not yet routine for several NRLs and are also in constant update, there is a need for NGS training. Since 2018, EURLs regularly organize training courses for their respective NRL network and the first joint training session with all the EURLs that are part of the WG was organized in 2022 focusing on dry lab analysis, with the aim of bringing NRLs together to ensure exchange of opinions and establishing fruitful collaborations. Moreover, a document [16] aimed at informing on training possibilities and an inventory of guidelines for both wet lab and dry lab NGS analyses was released, listing training events and hyperlink support for training courses created by EURLs or by companies providing either sequencing technologies (e.g. Illumina, Nanopore) or software for bioinformatic analyses [e.g. Bionumerics, SeqSphere, Center for Genomic Epidemiology (CGE)].

## Conclusions

This initiative has brought the EURLs together, allowing common strategies to be found to stimulate the adoption of NGS for monitoring microbiological foodborne pathogens. In 5 years of activity, the WG has released guidance documents for assisting NRLs in all the steps of NGS, helping the transition from classical molecular methods towards genomics while ensuring harmonization. These resources are routinely updated on EURL websites, making them available for any laboratory wishing to follow the same path, with the aim of contributing to improve preparedness in the use of NGS to characterize microbial hazards and trace the sources of infection.

## References

[1] The European Parliament and the Council of the European Union (2017). REGULATION (EU) 2017/625 OF THE EUROPEAN PARLIAMENT AND OF THE COUNCIL of 15 March 2017 on official controls and other official activities performed to ensure the application of food and feed law, rules on animal health and welfare, plant health and plant protection products, amending regulations (EC) no 999/2001, (EC) no 396/2005, (EC) no 1069/2009, (EC) no 1107/2009, (EU) no 1151/2012, (EU) no 652/2014, (EU) 2016/429 and (EU) 2016/2031 of the European Parliament and of the Council, Council Regulations (EC) No 1/2005 and (EC) No 1099/2009 and Council Directives 98/58/EC, 1999/74/EC, 2007/43/EC, 2008/119/EC and 2008/120/EC, and repealing Regulations (EC) No 854/2004 and (EC) No 882/2004 of the European Parliament and of the Council, Council Directives 89/608/EEC, 89/662/EEC, 90/425/EEC, 91/496/EEC, 96/23/EC, 96/93/EC and 97/78/EC and Council Decision 92/438/EEC. Official Journal of the European Union.

[2] European Commission Directorate General for Health and Food Safety (2019). Request for the implementation of a One Health system for the collection and analysis of whole-genome sequencing (WGS) datafrom human and food/animal isolates. https://open.efsa.europa.eu/study-inventory/EFSA-Q-2020-00101.

[3] ISO Technical Committee ISO/TC 34/SC 9 Microbiology (2022). ISO 23418:2022 Microbiology of the food chain — Whole genome sequencing for typing and genomic characterization of bacteria — General requirements and guidance. https://www.iso.org/standard/75509.html.

[4] Inter-EURLs Working Group on NGS (2020). Survey on the use of NGS across the NRLs networks. https://www.iss.it/documents/5430402/0/Survey+on+the+use+of+NGS+Del8.pdf/27f19f4b-1d85-8437-6ef0-a65aac2c6617?t=1614771657671.

[5] EURL for Escherichia coli including Shiga toxin-producing E. coli (2018). Inter-European Union Reference Laboratories Working Group on Next Generation Sequencing. https://www.iss.it/en/-/inter-european-union-reference-laboratories-working-group-on-next-generation-sequencing-1.

[6] COMPARE (2015). COMPARE EUROPE. https://www.compare-europe.eu/.

[7] Rene S. Hendriksen SKP, Leekitcharoenphon P, Malorny B, Borowiak M, Battisti A (2018). Final Report of ENGAGE - Establishing Next Generation Sequencing Ability for Genomic Analysis in Europe.

[8] Llarena A-K, Ribeiro-Gonçalves BF, Silva DN, Halkilahti J, Machado MP (2018). INNUENDO: A cross-Sectoral platform for the integration Ofgenomics in the surveillance of food-borne pathogens. EFSA supporting publications, EXTERNAL SCIENTIFIC REPORT. EFSA Supporting Publications.

[9] Inter-EURLs Working Group on NGS (2021). Guidance document for WGS-laboratory procedures. https://www.iss.it/documents/5430402/0/Guidance+document+for+WGS%20laboratory+procedures.pdf/2d0f3edb-7a46-754e-a0b3-2c4612b27de8?t=1637739547899.

[10] Inter-EURLs Working Group on NGS (2022). Supporting document for preparing high quality DNA for WholeGenome sequencing. https://www.iss.it/documents/20126/6704161/Del9_guidelines_DNAquality_forPublication_231%2022022.pdf/ba573c2e-aa9f-9f4b-f1fe-933186c92ee6?t=1672125522440.

[11] Inter-EURLs Working Group on NGS (2022). Bioinformatics tools for basic analysis of next generation sequencing data. https://www.iss.it/documents/20126/6704161/Del4_Tools_for_basic_analysis_forPublication_23%20122022.pdf/0def662e-0195-8af2-c956-ff26d09e7ae6?t=1672125015933.

[12] Inter-EURLs Working Group on NGS (2022). Guidance document for cluster analysis of whole genome sequence data. https://www.sva.se/media/8da546180a1a226/del5_guidelines_cluster_analysis_wg_ngs.pdf.

[13] Inter-EURLs Working Group on NGS (2020). Overview of conducted and planned proficiency tests. https://www.eurl-ar.eu/CustomerData/Files/Folders/34-%20wgs/587_del1-overview-of-pts-v1-161220-corrected-002.pdf.

[14] Inter-EURLs Working Group on NGS (2022). Reference WGS collection. https://www.eurlsalmonella.eu/sites/default/files/2023-03/Del2_Ref_Collection_v02Final_20230306.pdf.

[15] Inter-EURLs Working Group on NGS (2021). Guidance document for WGS-Benchmarking. https://sitesv2.anses.fr/en/system/files/NGS%20EURL%20WG%20-%20Del5%20-%20Benchmarking.pdf.

[16] Inter-EURLs Working Group on NGS (2022). Inventory of training supports. https://sitesv2.anses.fr/en/system/files/Del7_Inventory%20training%20supports%20for%20NGS%20_15-12-22_0.pdf.

